# Role of Nrf2, STAT3, and Src as Molecular Targets for Cancer Chemoprevention

**DOI:** 10.3390/pharmaceutics14091775

**Published:** 2022-08-25

**Authors:** Haseeb Ahsan, Salman Ul Islam, Muhammad Bilal Ahmed, Young Sup Lee

**Affiliations:** 1 Department of Pharmacy, Faculty of Life and Environmental Sciences, University of Peshawar, Peshawar 25120, Pakistan; 2 Department of Pharmacy, CECOS University, Peshawar 25000, Pakistan; 3 BK21 FOUR KNU Creative BioResearch Group, School of Life Sciences, Kyungpook National University, Daegu 41566, Korea

**Keywords:** Nrf2, STAT3, Src, NF κB, chemoprevention, inflammation

## Abstract

Cancer is a complex and multistage disease that affects various intracellular pathways, leading to rapid cell proliferation, angiogenesis, cell motility, and migration, supported by antiapoptotic mechanisms. Chemoprevention is a new strategy to counteract cancer; to either prevent its incidence or suppress its progression. In this strategy, chemopreventive agents target molecules involved in multiple pathways of cancer initiation and progression. Nrf2, STAT3, and Src are promising molecular candidates that could be targeted for chemoprevention. Nrf2 is involved in the expression of antioxidant and phase II metabolizing enzymes, which have direct antiproliferative action as well as indirect activities of reducing oxidative stress and eliminating carcinogens. Similarly, its cross-talk with NF-κB has great anti-inflammatory potential, which can be utilized in inflammation-induced/associated cancers. STAT3, on the other hand, is involved in multiple pathways of cancer initiation and progression. Activation, phosphorylation, dimerization, and nuclear translocation are associated with tumor cell proliferation and angiogenesis. Src, being the first oncogene to be discovered, is important due to its convergence with many upstream stimuli, its cross-talk with other potential molecular targets, such as STAT3, and its ability to modify the cell cytoskeleton, making it important in cancer invasion and metastasis. Therefore, the development of natural/synthetic molecules and/or design of a regimen that can reduce oxidative stress and inflammation in the tumor microenvironment and stop multiple cellular targets in cancer to stop its initiation or retard its progression can form newer chemopreventive agents.

## 1. Introduction

Cancer chemoprevention is the use of various means (natural and synthetic) to suppress, prevent, or delay cancer by inhibiting tumor development at the initial stages or by retarding the rate at which it grows to delay the malignant tendencies and properties of tumors [1,2].

At the cellular level, chemoprevention involves the halt or retardation of various molecular pathways at any or all three stages of cancer, i.e., initiation, promotion, and progression [3]. Such agents may block DNA damage during initiation or reduce free radical-induced damage. Furthermore, their potent antioxidant activities and ability to repair DNA may be additional mechanisms of chemoprevention [4]. Chemopreventive agents can also have profound effects on the progressive stages of cancer owing to their antiproliferative, anti-angiogenic, and antiapoptotic effects [3,5,6]. These actions are exerted by affecting various cellular signaling pathways, such as Nrf2, NF-κB, STAT3, and Src [5,7,8].

## 2. Nuclear Factor E2-Related Factor 2 (Nrf2)

### 2.1. Role and Significance of Nrf2 in Cancer

**Figure 1:**
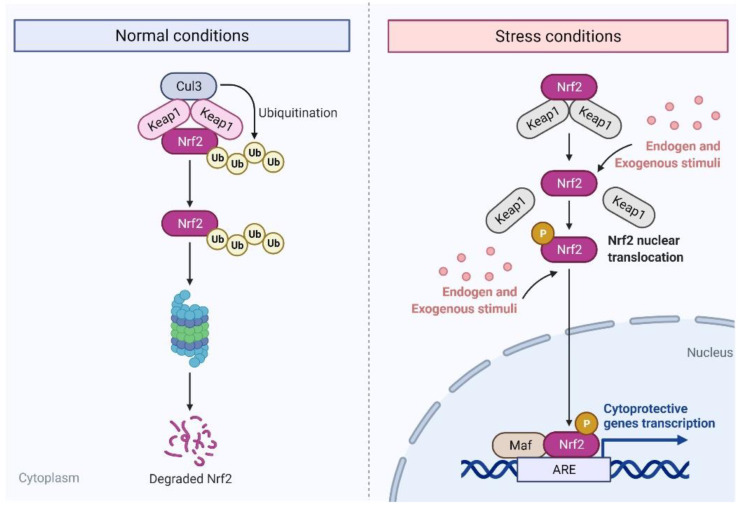
States of Nrf2 in normal conditions and under stress by various stimuli. ARE, antioxidant response element; Keap1, Kelch-like ECH-associated protein 1; Maf, avian musculoaponeurotic fibrosarcoma.

Nrf2 is an important transcription factor that regulates cancer gene expression and inflammation [9]. In the resting or inactive state, it binds to Keap1. In the bound state, it undergoes constant ubiquitination by Cullin 3-dependent E3 ubiquitin ligases. When activated, Keap1 undergoes modification, and Nrf2 is released from the complex. It enters the nucleus, dimerizes with the Maf proteins, and binds to the antioxidant response elements of the target genes [10] (Figure 1). Activation of Nrf2 may occur via MAPK, such as p38 and JNK [11]. Nrf2 is responsible for the expression of many enzymes involved in phase II metabolism (making xenobiotics more water soluble and readily excretable) and protects cells from oxidative damage. These enzymes include γ-glutamylcysteine synthetase (γGCS), NADPH quinone oxidoreductase 1 (NQO1), and heme oxygenase 1 (HO-1) [12]. The γ-GCS is involved in the biosynthesis of glutathione, which is one of the major antioxidants in human cells. NQO1 is responsible for generating reduced forms of ubiquinone and tocopherols, making them effective in eliminating free radicals. HO-1 plays a significant antioxidant role in maintaining cellular homeostasis against reactive oxygen species (ROS) [13].

### 2.2. Targeting Nrf2 Signaling for Cancer Chemoprevention

**Figure 2:**
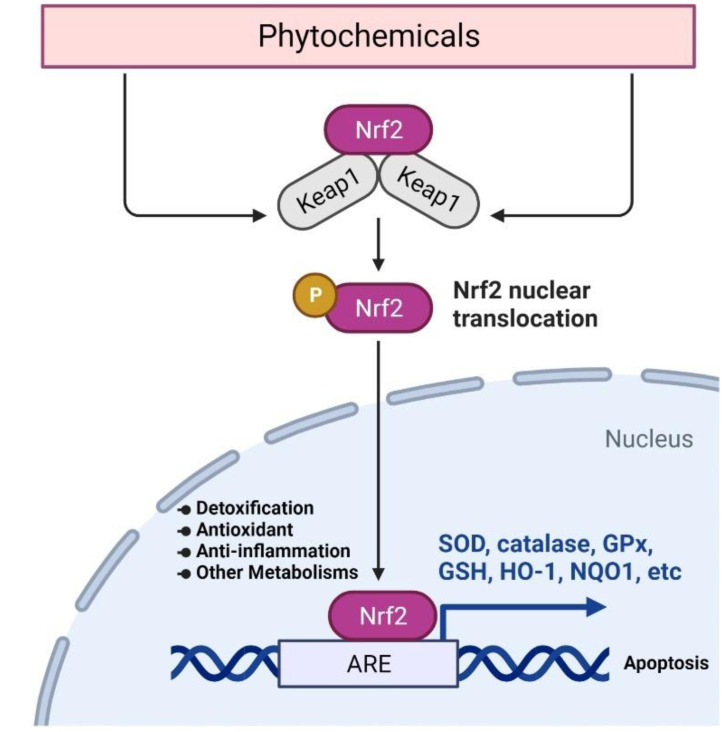
General mechanism of Nrf2-mediated expression of various detoxification and antioxidant enzymes by potential chemoprevention agents such as phytochemicals.

Nrf2 is an effective molecular target to induce chemopreventive effects. Many activators of the Nrf2 pathway have been shown to enhance the defensive capacity of cells against cancer, inflammation, and oxidative damage (Figure 2) [14,15,16,17].

A study by Lida et al. (2007) showed that Nrf2 null mice are more susceptible to developing chemically induced carcinoma of the urinary bladder [18,19]. Ginnalin A, a natural phenolic compound, has been reported to possess chemopreventive properties in human colon cancer via the activation of Nrf2 signaling. This study revealed that the compound suppressed the proliferation of cancer cells by arresting the cell cycle in the S phase. The compound also increased the translocation and expression of Nrf2, along with other antioxidant genes such as HO-1 and NQO1 [20]. Lycopene, a phytoconstituent in tomatoes, has been reported to attenuate the formation of tumors and their proliferative capacity. Lycopene was found to stimulate nuclear translocation of Nrf2 and the expression of various antioxidant enzymes. The study also reported that the release of Nrf2 from the complex was due to the enhanced expression of p62, which caused degradation of Keap1 [21]. Miconazole has also been reported to increase the expression of autophagy protein p62 in bladder carcinoma. The expression of p62 directly correlates with Nrf2 activation and decreases Keap1 expression [22].

### 2.3. Correlation between Nuclear Factor κB (NF κB) and Nrf2 Expression in Cancer

NF-κB is a transcription factor involved in regulating inflammation and cell proliferation [23]. It is also believed to serve as a link between inflammation and cancer [24]. In the resting state, it binds to the cytoplasmic IκBs. Proteasomal degradation of IκBs induced through their phosphorylation by kinases causes the release of NF-κB, its translocation to the nucleus, binding to specific response elements, and influences gene expression [25]. It also increases the expression of IL6, IL1β, TNFα, COX2, iNOS, and other cytokines. Furthermore, genes affected by NF-κB are involved in cell proliferation, angiogenesis, and metastasis [23,26,27]. NF-κB has also been linked to the emergence of resistance to endocrine therapy in breast cancer, suggesting that modulation of inflammation via this pathway is a significant requirement for cancer management [28]. Therefore, abnormal activation of this pathway may lead to the transition from inflammation to tumorigenesis. Likewise, the inhibition of inflammation may serve as an important mechanism to prevent cancer.

**Figure 3:**
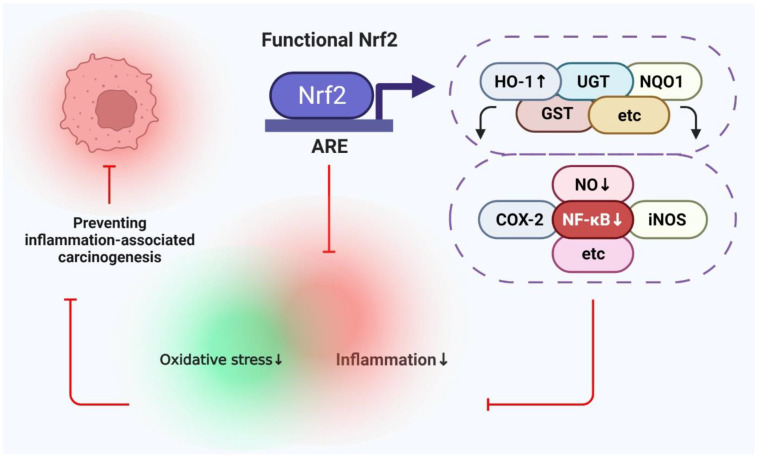
Correlation between Nrf2 and NF-κB in counteracting inflammation-associated cancer. Activation of Nrf2 after binding to antioxidant response element (ARE) leads to the production of antioxidant enzymes (HO-1, UGT, NQO1, GST), which can lead to suppression of NF-κB. This leads to indirect inhibition of NF-κB targets enzymes such as NO, COX-2, and iNOS. The overall effect is a reduction in oxidative stress and inflammation by the Nrf2 pathway, which can serve as a valuable target in preventing inflammation-associated cancer.

Nrf2 has been employed as a target for designing anti-inflammatory drugs for various chronic disorders such as multiple sclerosis. Dimethyl fumarate, used as a disease-modifying agent in multiple sclerosis, targets Nrf2. This agent is used because of its potent anti-inflammatory effect, which causes symptomatic relief in multiple sclerosis [29]. One study investigated the effects of sappanone A (*Caesalpinia sappan*) in macrophages. Sappanone A reduces nitric oxide (NO), prostaglandin E2 (PGE2), and IL-6. Isoflavanone also protects C57BL/6 mice from LPS-induced oxidative damage. Sappanone increased the mRNA expression of Nrf2 and its target genes NQO1 and HO-1. This study demonstrated that knockdown of Nrf2 inhibited the induction of HO-1 by sappanone A. Sappanone A also reduced LPS-induced NF-κB activation. The authors concluded that sappanone A exerts its anti-inflammatory effects by regulating Nrf2 and NF-κB signaling. This study links these two pathways, which can be extrapolated to the connection between inflammation and cancer [30]. Another study identified a sesquiterpene coumarin strigoid that could induce Nrf2 and suppress NF-κB simultaneously. Hence, such natural organic compounds may also be molecules of interest that can target both pathways to suppress inflammation in cancer effectively. Moreover, they could prove to be effective chemopreventive agents as monotherapy or combination therapy [31]. Another study evaluated the effects of xanthohumol and phenethyl isothiocyanate on pancreatic cancer cells. The combination of both agents had better anti-inflammatory and anticancer properties than the individual agents. A combination of the two inhibited the binding of NF-κB to DNA by 47–60%. Other effects observed included enhanced expression of Nrf2, NQO1, and superoxide dismutase (SOD). Thus, the combination reduced oxidative damage, angiogenesis, and proliferation of pancreatic cells. This study suggests the use of these two agents as good chemopreventive agents to delay the progressive stages of cancer [32]. Moringa isothiocyanate 1 increases the nuclear accumulation of Nrf2 and reduces the nuclear translocation of NF-κB, both leading to a reduction in inflammation and oxidative stress [33]. Another study reported that Nrf2-mediated stimulation of antioxidant pathways causes a reduction in ROS, which is a stimulant for NF-κB activation. Hence activation of Nrf2 leads to suppression of NF-κB [34]. Therefore, it is pertinent to mention that agents targeting Nrf2 and NF-κB can prove to be a very useful means of chemoprevention by targeting the tumor directly and inhibiting chronic inflammation associated with cancer [26,35]. The same correlation between Nrf2 and NF-κB is shown in Figure 3.

### 2.4. Targeting Nrf2 in Colitis-Associated Colon Cancer

Colon cancer is a huge health burden, as it is one of the most commonly diagnosed cancers in both men and women [36,37]. It has also been reported that chronic inflammation of the gastrointestinal tract due to infections, aberrant immune responses, or environmental factors can promote the progression of colon cancer [38].

In inflammatory bowel disease (IBD), the luminal side of the epithelial cells is disrupted by inflammation of the intestines by cytokines and chemokines [39]. Continuous and long-term severe IBD increases the risk of colorectal cancer [40,41]. Certain proinflammatory cytokines, such as IL-23, produced in excessive quantities in IBD, can also lead to the aggravation of colon cancer [42,43,44]. Various studies have reported that inflammation of the gastrointestinal tract in various forms helps in the progression of cancer [45,46]. In experimental settings, IBD can be chemically induced in murine models using dextran sodium sulfate (DSS). DSS is toxic to epithelial cells, causes erosion, and reduces membrane integrity [47,48]. The known cause of epithelial damage due to DSS is the overproduction of ROS [49,50,51]. Nrf2 regulates the expression of several cellular antioxidant enzymes to counteract the effects of ROS [52]. It has been shown experimentally that Nrf2 null mice have a greater tendency to develop DSS-induced IBD than wild-type mice. Moreover, the levels of inflammatory cytokines were higher in Nrf2 null mice. This greater propensity for epithelial damage is attributed to a reduction in phase II detoxifying enzymes, whose expression is regulated by Nrf2 [53,54]. It has been postulated that inflammation is one of the prominent characteristics of the tumor microenvironment in colon cancer [55,56]. A study demonstrated that Nrf2 knockout mice have a greater number and size of tumors induced by Azoxymethane or DSS. It reported that the knockout mice had 80% adenocarcinoma lesions compared to 29% in wild type. This shows that Nrf2 exerts a protective role in colitis-associated colon cancer [56].

Many phytoconstituents have been shown to exert beneficial effects in chemically induced colitis through Nrf2 activation. One study demonstrated that the pretreatment of C57BL/6 mice with the isothiocyanate compound sulforaphane (25 mg/kg) reduced the severity of DSS-induced colitis. The loss of body weight and disease activity index were lower than those in the untreated group. The pretreated group also had a longer colon, reduced expression of proinflammatory markers, and increased expression of Nrf2-related genes [57]. Another study has reported the effects of coenzyme Q10 on colitis. Researchers found that Q10 can potentially protect the colon in an Nrf2-dependent manner. They reported that the suppression of colitis and a reduction in the levels of inflammatory markers are due to activation of the Nrf2-dependent HO-1 pathway [58]. A study also demonstrated that the use of agents that cause the breakdown of the Nrf2-Keap1 complex and facilitate its nuclear translocation in colon cells (NCM460) exerted cytoprotective effects against ulcerative colitis. In this study, CPUY192018 was used as an inhibitor of the Nrf2-Keap1 complex [52]. 

## 3. Signal Transducers and Activators of Transcription-3 (STAT3)

### 3.1. Significance of STAT3 

**Figure 4:**
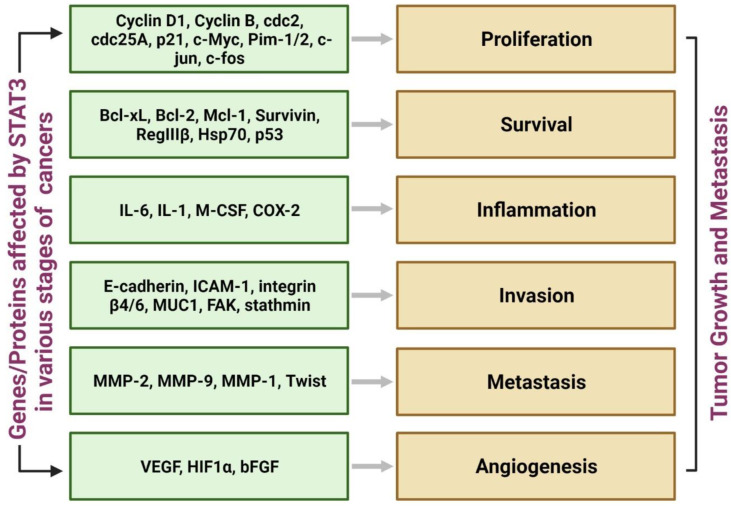
Complex Role of STAT3 in tumorigenesis and its progression.

STAT3 is one of the main regulators of the cell cycle and is involved in its differentiation, proliferation, apoptosis, and angiogenesis [59,60]. Cancer cells undergoing rapid proliferation require persistent STAT3 activation. In gastric carcinoma, IL-26-induced abnormal STAT3 activation causes upregulation of the antiapoptotic proteins, such as Bcl2, which leads to uncontrolled cell proliferation [61]. Its persistent activation has also been associated with endometrial cell proliferation in uterine cancer [62]. The same has been reported for bladder, colon, and renal carcinoma [63,64]. Cancer cells can speed up glycolysis while downregulating mitochondrial respiration (Kreb’s cycle). This leads to a greater conversion of pyruvic acid to lactic acid (Warburg effect). Increased lactate is indicative of hypoxia leading to induction of HIF-1a. It induces pyruvate kinase leading to greater pyruvate production for further conversion to lactate and consequent HIF-1a release. Pyruvate kinase induction has been associated with greater STAT3 activation [65]. STAT3 is responsible for transcriptional activation of the VEGF gene. It is also associated with increased expression of MMP-2 and VEGF, which are associated with the increased invasive and metastatic transformation of cancers [66,67]. It plays a significant role in G1 -S phase transition by upregulation of cyclin D1, Cdc 25A, and downregulation of p21. STAT3 activation leading to change in expressions and concentrations of various cytokines and transcription factors leading to invasive carcinogenesis is summarized in Figure 4.

### 3.2. Strategies to Target STAT3 

Chemoprevention strategies can be devised using STAT3 as a target molecule. The persistent activation of STAT3 in cancer can be counteracted by various mechanisms such as (i) inhibition of receptors leading to STAT3 activation; (ii) inhibition of ligand binding to STAT3 activating receptor; (iii) inhibition of the phosphorylation of the cytoplasmic tail of the receptor; (iv) inhibition of JAK kinases to cease STAT3 dimerization; and (v) prevention of its nuclear translocation and binding to specific response elements on DNA [68,69,70]. 

STAT3 is also a downstream cellular mediator of cancer and angiogenesis, induced by IL-6 and EGFR. Various tyrosine kinase inhibitors (AG490 and AZD1480) are currently being studied to block the JAK-STAT3 pathway, thereby inhibiting tumorigenesis and angiogenesis [71,72]. Novel compounds that can block STAT3 dimerization can also be effective chemopreventive agents. Garcinol, a natural compound isolated from *Garcinia indica*, has been reported to suppress STAT3 signaling in hepatocellular cancer. The mechanism of STAT3 inhibition involves the binding of garcinol to the SH2 domain of STAT3 and inhibition of its dimerization. Additionally, it inhibits STAT3 acetylation, leading to impaired binding to DNA. This results in the suppression of many target genes involved in cell proliferation and angiogenesis. Moreover, an increase in apoptosis was observed. Hence, garcinol and its semisynthetic derivatives may be effective future treatments for chemoprevention and chemotherapy [73]. A recently published study has reported the effects of STAT3 inhibition by ODZ10117. The molecule inhibits dimerization by binding to its SH2 domain. The net result observed was inhibition of phosphorylation and nuclear translocation. This inhibitory effect was stronger than other known STAT3 inhibitors such as STA-21. ODZ10117 suppresses tumor cell migration. It also induced apoptosis and reduced cell invasiveness. Overall, this molecule and its analogs can potentially be used as chemopreventive agents that can delay cancer progression [74]. The functional phosphorylation sites in STAT3 are tyrosine 705 and serine 727. Most natural and synthetic inhibitors discovered/developed block STAT3 phosphorylation at tyrosine 705. A summary of inhibitors of STAT3 phosphorylation is given in Table 1.

### 3.3. Chemopreventive Agents Targeting STAT3

Guggulsterone, isolated from *Commiphora mukul*, has also been reported to possess anticancer potential. It induces apoptosis and causes cell cycle arrest. The combination regimen increased the antineoplastic effects of erlotinib, cetuximab, and cisplatin in squamous cell carcinoma of the head and neck. Guggulsterone has been found to reduce the expression of STAT3 and induce apoptosis [75,76]. Another study reported that guggulsterone causes a decrease in the levels of phosphotyrosine STAT3 in multiple myeloma and squamous cell carcinoma. Furthermore, it also inhibits LPS-induced inflammatory cytokines in the NF-κB pathway [77]. A synthetic derivative of guggulsterone, GSD-1, has recently been reported to exert its strong inhibitory effects on NF-κB which helped reduce the metastatic potential of breast cancer cells [78].

Astaxanthin, a ketocarotenoid produced by certain algae, has been reported to block DMBA-induced hamster buccal pouch (HBP) carcinomas by downregulating JAK/STAT signaling. Astaxanthin has been found to reduce the expression of genes involved in the JAK/STAT3 pathway, such as cyclin D1, MMP-2, and VEGF. Thus, it reduces tumor cell proliferation, invasion, and angiogenesis [79]. 

Curcumin is a natural polyphenolic present in turmeric rhizome. It has been reported to suppress STAT3 and NF-κB signaling. A study demonstrated that a combination of epigallocatechin gallate and curcumin suppresses STAT3 phosphorylation in breast cancer-derived stem cells. It also reduces COX-2 activity and, therefore, can be considered a good agent for combing with those potentiating Nrf2 antioxidant pathways. It also increases cancer cell apoptosis mediated by Bcl-2. Proapoptotic effects are more pronounced when used with Wnt signaling inhibitors [80].

Silibinin is a natural flavonoid with chemopreventive potential. Silibinin has been reported to have anti-inflammatory and antineoplastic properties. In the current study, silibinin significantly inhibited the viability of intestinal tumor cells. The production of inflammatory cytokines and phosphorylation of STAT3 is inhibited in intestinal tumor cells. Silibinin (750 mg/kg) decreased the number and size of tumors induced by azoxymethane/DSS. Colitis and tumor scores decreased. The rate of proliferation also reduced with an increase in tumor cell apoptosis. Moreover, silibinin reduced the production of inflammatory cytokines and attenuated the impairment of the colonic mucosal barrier. Furthermore, an interaction of the probe with cellular molecules showed that silibinin suppressed the LPS-induced upregulation of STAT3 phosphorylation. It also reduced the expression of IL-6. The study concluded that silibinin had chemoprotective potential via the IL-6/STAT3 pathway, giving it dual beneficial activity in cancer and inflammation, both of which are present in colitis-associated cancer [81].

Acetoside is a naturally occurring glycoside found in plants. Its chemopreventive potential was assessed in a rat model of hepatocellular cancer chemically induced by diethyl nitrosamine (DEN). Acetoside was administered at 0.1% and 0.3% of their diet 2 weeks before chemical induction of hepatic cancer. Treatment was continued for 18 weeks. Histological studies have shown that acetoside reduces nodule size in hepatocellular cancer. The levels of biochemical markers of hepatocytic injury (ALT), inflammation (IL-6, IFN-γ, and TNF-α), and apoptosis (Caspase-3) improved after the administration of acetoside. It also ameliorated the DEN-induced DNA damage, cytotoxicity, and genotoxicity. It also considerably reduced oxidative damage. Furthermore, the reduced expression of NF-κB, Bcl2, and STAT3 showed anti-inflammatory, proapoptotic, and anticancer potential. Thus, acetoside induced STAT3-mediated antioxidant and anti-inflammatory effects along with its antiproliferative and proapoptotic properties, making it an agent with good chemopreventive potential [82].

### 3.4. Targeting STAT3 Improves Sensitivity of Other Anticancer Agents

Since STAT3 is the converging point of many upstream stimuli, receptors, and ligands, combining anti-STAT3 agents with other chemotherapy or immunosuppressants might offer promising ways to delay the progression of aggressive cancers. The STAT3 pathway is involved in the pathogenesis of EGFR-dependent squamous cell carcinoma (SCC). The invasive potential of EGFR-mediated SCC is greatly enhanced by the persistent activation of STAT3. A previous study suggested that combining anti-EGFR and anti-STAT3 agents would prove a practically effective mode of cancer suppression [83]. Since SCC and other solid tumors are resistant to anticancer agents, their suppression and elimination may warrant blocking via multiple pathways [84]. It has also been reported that SCC of the head and neck is more sensitive to cetuximab when combined with a short hairpin RNA knockdown approach for STAT3 inhibition. Combination therapy enhances DNA damage and apoptosis in cancer cells, as STAT3 activation is important for cell survival [83].

In pancreatic cancer, STAT3 activation has also been correlated with developing resistance to MEK inhibitors. Resistance is due to mutations in K-Ras and MEK inhibitors that target the Ras pathway and are less efficacious. The authors reported that the use of MEK inhibitors such as AZD6244 and trametinib caused profound activation of STAT3 in K-Ras mutant pancreatic cancer. We believe that STAT3 may be an important factor in developing resistance to MEK inhibitors in K-Ras-mutated pancreatic cancer. Therefore, a combination of LY5 (STAT3 inhibitor) and trametinib (MEK inhibitor) was administered to assess anticancer efficacy in resistant cancer. The results showed that trametinib displayed better tumor suppression in the presence of a STAT3 inhibitor. The authors concluded that the STAT3 regimen improves the efficacy of anti-MEK agents in resistant pancreatic cancers [85].

Curcumin has been reported to have a good chemopreventive potential. One known mechanism is the suppression of the JAK/STAT3 pathway. Epigallocatechin gallate is another promising candidate as a chemopreventive agent for cancer. This study assessed the effect of the two as monotherapy and combination therapies on angiogenesis in colorectal carcinoma cell lines (HCT116 and HT-29). Although both inhibited angiogenesis via inhibition of JAK/STAT3 signaling, their individual effects were minimal. However, the anti-angiogenic effects were potentiated when a combination of the two was used [86,87]. 

To devise new therapeutic strategies for cholangiocarcinoma, the combined effects of doxorubicin and β-caryophyllene were studied in Mz-ChA-1 and H69 cholangiocyte cell lines. In the carcinoma cell line Mz-ChA-1, β-caryophyllene synergized with the cytotoxic effect of doxorubicin at lower doses. However, it exerted cytoprotective effects on the H69 cell line (non-malignant cholangiocytes) after exposure for 24 h. Mechanistic insights revealed that the synergistic cytotoxicity of doxorubicin was due to cell cycle arrest in the G2/M phase by β-caryophyllene. It was also observed that the presence of β-caryophyllene improved the suppression of STAT3 signaling by doxorubicin. Hence, this study reports that the sensitivity of cholangiocarcinoma to doxorubicin is improved due to better suppression of STAT3 signaling by the concomitant use of β-caryophyllene, making it a good candidate as a chemosensitizer and chemopreventive agent [88].

**Table 1:** Various natural and synthetic STAT3 antiphosphorylating agents.

	Inhibitor of STAT3 Phosphorylation	Class of Compound	Mechanism	References
1	Alantolactone	Sesquiterpene lactone	Binds and inhibits phosphorylation at Tyr705 in pancreatic cancer	[89]
2	S-3I 1757	Synthetic; salicylic acid derivative	Binds pTyr 705 at SH2 domain; inhibits dimerization	[90]
3	B12	Synthetic; sulfamoyl benzamide derivative	Inhibits phosphorylation at Tyr 705; inhibits STAT3 phosphorylation induced by IL-6	[91]
4	Cinobufagin	Natural; bufadienolide	Inhibits STAT3 phosphorylation; Inhibits EMT; Inhibits IL-6 mediated STAT3 translocation in colon cancer	[92]
5	ACT001	Synthetic; parthenolide derivative (sesquiterpene lactone)	Directly binds STAT3, inhibits phosphorylation; inhibits PD-L1 in glioblastoma	[93]
6	Resveratrol	Natural stilbenoid	Inhibits IL-6 induced phosphorylation at Tyr 705; inhibits EMT in cervical cancer	[94]
7	Piperine & piperlongumine	Natural alkaloids	Combination inhibits STAT3 phosphorylation; induces apoptosis selectively in breast cancer cells	[95]
8	Curcubitacin B	Natural triterpene	Inhibits STAT3 phosphorylation at Tyr 705; inhibits its nuclear translocation; induces apoptosis in gastric cancer	[96]
9	oleacein	Natural polyphenolic	Reduces cell adhesion, migration, inhibits STAT3 phosphorylation; induces apoptosis in neuroblastoma	[97]
10	HJC0152	Synthetic niclosamide derivative	Inhibits STAT3 phosphorylation at Tyr 705; reduces glutamine and glutathione causing oxidative stress-mediated apoptosis in lung cancer.	[98]
11	Costunolide	Natural sesquiterpene	Inhibits STAT3 phosphorylation at Tyr 705; inhibits metastasis in osteosarcoma	[99]
12	Ginsenoside Rh1	Natural triterpenoid saponin	Inhibits its phosphorylation, nuclear translocation, and accumulation; inhibits NF-κB in triple-negative breast cancer	[100]
13	Convollatoxin	Natural glycoside	Inhibits STAT3 phosphorylation at Tyr 705 & Ser 727; inhibits phosphorylation of JAK1, JAK2 & Src; promotes apoptosis in colon cancer	[101]
14	SS-4	Synthetic Phenoxyacetamide derivative	Inhibits STAT3 phosphorylation at Tyr 705; highly potent and STAT3 selective glioblastoma tumor growth inhibitor	[102]
15	WZ-2-033	Synthetic acetamide derivative	Inhibits STAT3 phosphorylation at Tyr 705; inhibits its dimerization & nuclear translocation; inhibits metastasis; induces apoptosis in triple-negative breast cancer and gastric cancer	[70]

## 4. Src

**Figure 5:**
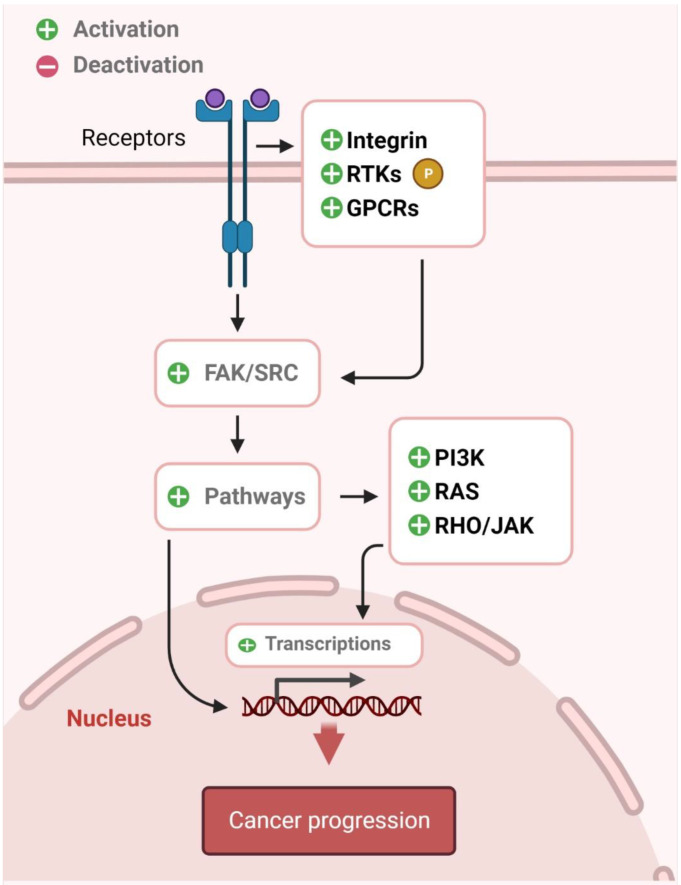
Upstream stimuli of Src and its downstream target molecules. Src interacts with integrin/focal adhesion kinase (FAK), the receptor of tyrosine kinases (RTKs), and G-protein-coupled receptors (GPCRs), leading to activation of its downstream target proteins, including MAPK/ERK, PI3K, IL-6/JAK/STAT3, and Rho/Rho-associated protein kinase (ROCK), subsequently promoting cancer progression.

Src was the first oncogene to be discovered [103]. It is a prototype of the Src family of kinases, which are non-receptor tyrosine kinases. These include Fyn, Yes, Blk, Yrk, Fgr, Hck, Lck, and Lyn. Src, Fyn, and Yes are ubiquitously expressed in many tissues. Higher concentrations have been observed in neurons, platelets, and osteoclasts [104]. Their basic function is to catalyze the transfer of phosphate from ATP to tyrosine residues at specific positions in proteins. This tyrosine phosphorylation causes activation and transduction of downstream molecules to transmit signals. The ultimate result of this signaling may be the activation of nuclear factors, resulting in gene expression or reorganization of the cell cytoskeleton [105]. Figure 5 shows the upstream stimuli of Src and its downstream pathway molecules, leading to cell survival, proliferation, and cancer progression.

### 4.1. Significance of Src in Cancer

**Figure 6:**
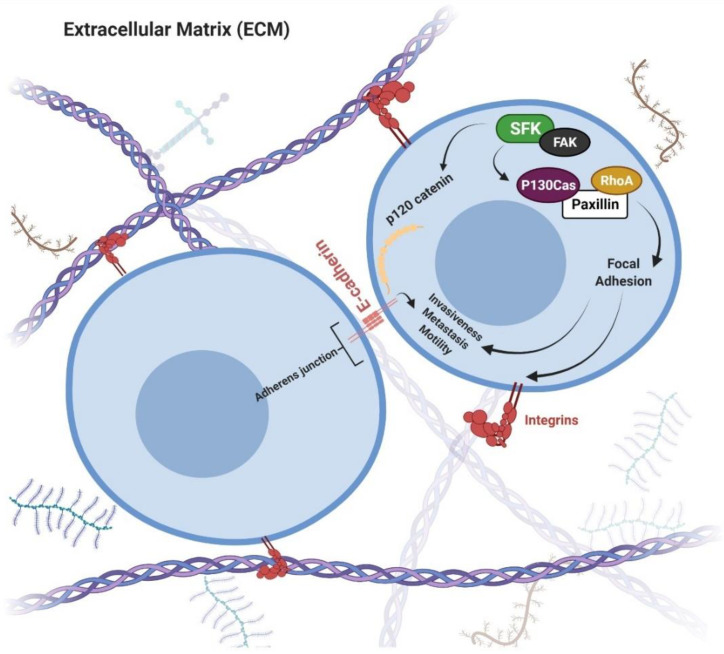
Activated SFKs can phosphorylate p120 catenin and FAK, causing destabilization of E-cadherin and adherens junction. The p130cas and paxillin are cell migration mediators. Src-FAK-p130cas axis causes cell invasion via MMP activation. All these cellular changes promote the invasiveness of cells.

Studies have shown that upon activation, Src induces cell growth and survival, leading to the promotion of tumor formation, promotion of reorganization of the cell actin cytoskeleton, and p120-mediated disruption of tight junctions, which subsequently facilitates the invasion and motility of cells (Figure 6) [106,107]. Src overactivation has been observed in multiple cancers, including melanoma, glioma, gastric, pancreatic, colorectal, prostate, breast, lung, head, and neck [108,109,110,111,112,113,114,115,116]. 

It has been shown that the migration and invasion of cancer cells underlie the ‘‘mesenchymal-type” mechanism where matrix-degrading proteases promote the locomotion of cancer cells through pericellular ECM breakdown [117]. Studies have shown that Src is involved in the increased expression of MMPs via diverse pathways, including the Src/Akt and NF-κB pathways [118], ERK and PI3K signaling [119], and PKC/MAPK/AP-1/LEF-1 and PI3K/AP-1/LEF-1 pathways [120]. Furthermore, Src has been reported to upregulate the expression of MMPs. A study reported that WNT5A upregulates Src, which induces MMP-14, all leading to invasion in osteosarcoma cells [121]. Hypoxia also enhances the invasive characteristics of malignant cells and angiogenesis via stimulation of the Src signaling pathway. Cannabidiol has been reported to inhibit Src, which causes a reduction in the hypoxia-inducible factor and consequent angiogenesis in breast cancer cells [122]. Additionally, Src has been shown to facilitate tumor cell extravasation by enhancing VEGF-induced vascular permeability [123].

Epithelial-mesenchymal transition (EMT) is a complicated process through which epithelial cells attain the characteristics of mesenchymal cells with increased migratory potential [124]. Loss of E-cadherin is a hallmark of EMT, in which cells detach from each other and start migrating to other parts of the body [105,125]. It has been shown that the activation of Src phosphorylates the E-cadherin–β-catenin complex, dissociating the latter, and subsequently induces EMT [126]. Upon dissociation from the complex, β-catenin translocates to the nucleus, where it transcriptionally activates various EMT-related targets such as Snail-family members, vimentin, Myc, matrix-degrading proteases, and cyclin D [127,128]. Src-induced EMT is related to and may promote the metastatic potential of cancer cells [129,130,131].

### 4.2. Chemoprevention via Src Inhibition

The inhibitory effect of apigenin on TGF-β-stimulated VEGF production in human prostate carcinoma cells was analyzed. The authors reported that apigenin blocked VEGF and TGF-β1-induced phosphorylation and was correlated with cancer progression, especially in Smad2/3 and Src/FAK/Akt signaling pathways [132]. It has been shown that activation of Src actively participates in early stage (ER-negative) breast cancer initiation, whereas downregulation of Src significantly inhibits cancer progression involving various underlying mechanisms such as suppression of Myc translation, reduction of GLUT1 transcription, and glucose uptake by tumor cells. These findings highlight the importance of Src inhibitors in the prevention and treatment of ER-negative breast cancer [133].

An investigation reported that shikonin blocked STAT3/FAK/Src signaling and stimulated the suppression of stem cell load in vitro, tumorigenicity, and metastasis in vivo. Compared to individual inhibitors, the combined blockade of STAT3 with Src or FAK decreased cell migration, invasion, and mammosphere formation more significantly [134]. S100 calcium binding proteinA7 (S100A7) is upregulated in many cancers and is associated with the facilitation of metastasis. A study reported that metastasis induced by S100A7 is reduced by flavonoids, such as luteolin and quercetin, via inhibition of Src/STAT3 signaling in A431-III cells. Flavonoids reduce the levels of S100A7, phosphorylated Src, and phosphorylated STAT3. The effect of flavonoids on EMT markers, such as E-cadherin, was also observed. Flavonoids increased the levels of E-cadherin and were found to resist metastasis of cancer cells in zebrafish larvae [135]. Another study reported that the extract of *Morus alba* could suppress the metastatic potential of non-small cell lung cancer cells. The plant extract also caused the downregulation of EMT markers such as Slug and vimentin. It also upregulates the expression of occludin, a tight junction protein. Insights into the anti-invasive and anti-metastatic mechanisms revealed that the plant extract decreased the activation of STAT3 and Src by inhibiting their phosphorylation [136].

## 5. Conclusions

Recently, chemoprevention studies have gained popularity because of the basic concept of delaying the production (if absent) or progression (if present) of cancer. Therefore, investigations leading to the discovery of molecular pathways and target cellular molecules are necessary for a complete understanding of chemopreventive mechanisms. The molecular targets for chemopreventive agents described in this review (Nrf2, STAT3, and Src) have shown to be promising candidates with chemopreventive potential in various cancer cell lines or carcinogen-induced tumorigenesis models in animals. Structural modifications of chemoprevention agents into safer and more effective chemoprevention agents are needed. Recently, researchers have synthesized such derivatives that include dimethylaminomethyl curcumin, diarylyheptanoid curcumin [137], and GSD-1. Moreover, effective delivery of therapeutic doses of medicaments requires careful design of drug delivery systems such as lipid-based nanoparticles. These modifications may enable the agent to effectively concentrate at its target sites and regulate the intracellular molecular targets (Nrf2, STAT3, etc.) to counteract cancer. Emphasis must be given to changing the chemical structures such that their aqueous and lipid solubility becomes optimal for optimal drug delivery and bioavailability. Additions of groups capable of binding specific proteins present in the tumor microenvironment may increase their selectivity toward tumor cells. The use of agents acting on Nrf2, STAT3, Src, and NF-κB, etc., can be looked into in various cancers aggravated by oxidative stress, such as estrogen-dependent breast cancer and steatohepatitis. Furthermore, the discovery and synthesis of agents which can act on Nrf2, STAT3, and NF-κB (like physodic and salazinic acids [138]) is yet another area to be explored for effective and timely chemoprevention.

## Data Availability

Not applicable.

## Abbreviations

Bcl-2	B-cell lymphoma 2
Bcl-xL	B-cell lymphoma-extra-large
bFGF	Basic fibroblast growth factor
c-Myc	Cellular myelocytomatosis
Cdc	Cell division cycle
COX-2	Cyclooxygenase-2
DSS	Dextran sodium sulfate
FAK	Focal adhesion kinase
GCS	Glutamylcysteine synthetase
HIF	Hypoxia-inducible factor
HO-1	Heme oxygenase 1
IBD	Inflammatory bowel disease
ICAM-1	Intercellular adhesion molecule-1
IL	Inter leukin
iNOS	Inducible nitric oxide synthase
IκB	Inhibitor of Nuclear factor-κB
JNK	C-Jun N-terminal kinase
LPS	Lipo polysaccharide
M-CSF	Macrophage colony-stimulating factor
Maf	Musculoaponeurotic fibrosarcoma
MAPK	Mitogen-activated protein kinase
MMP	Matrix metalloproteinase
MUC1	Mucin 1
NF-κB	Nuclear factor κB
NO	Nitric oxide
NQO1	NADPH quinone oxidoreductase 1
Nrf2	Nuclear factor Erythroid factor 2-related factor 2
PGE_2_	Prostaglandin E_2_
PI3K	phosphoinositide 3-kinase
ROS	Reactive oxygen species
RTK	receptor tyrosine kinase
SCC	Squamous cell carcinoma
SFK	Src family kinase
SOD	Superoxide dismutase
STAT3	Signal Transducers and Activators of Transcription-3
TNF	Tumor necrosis factor
VEGF	Vascular endothelial growth factor
